# Evaluating the impact of type 2 diabetes mellitus on pulmonary vascular function and the development of pulmonary fibrosis

**DOI:** 10.3389/fendo.2024.1431405

**Published:** 2024-07-10

**Authors:** Nhlakanipho Mzimela, Nosipho Dimba, Aubrey Sosibo, Andile Khathi

**Affiliations:** Department of Human Physiology, Faculty of Health Sciences, University of KwaZulu-Natal, Durban, South Africa

**Keywords:** type 2 diabetes mellitus (T2DM), prediabetes, pulmonary vascular function, pulmonary vascular dysfunction, pulmonary fibrosis

## Abstract

The increasing prevalence of type 2 diabetes mellitus (T2DM) is a significant worldwide health concern caused by sedentary lifestyles and unhealthy diets. Beyond glycemic control, T2DM impacts multiple organ systems, leading to various complications. While traditionally associated with cardiovascular and microvascular complications, emerging evidence indicates significant effects on pulmonary health. Pulmonary vascular dysfunction and fibrosis, characterized by alterations in vascular tone and excessive extracellular matrix deposition, are increasingly recognized in individuals with T2DM. The onset of T2DM is often preceded by prediabetes, an intermediate hyperglycemic state that is associated with increased diabetes and cardiovascular disease risk. This review explores the relationship between T2DM, pulmonary vascular dysfunction and pulmonary fibrosis, with a focus on potential links with prediabetes. Pulmonary vascular function, including the roles of nitric oxide (NO), prostacyclin (PGI2), endothelin-1 (ET-1), thromboxane A2 (TxA2) and thrombospondin-1 (THBS1), is discussed in the context of T2DM and prediabetes. Mechanisms linking T2DM to pulmonary fibrosis, such as oxidative stress, dysregulated fibrotic signaling, and chronic inflammation, are explained. The impact of prediabetes on pulmonary health, including endothelial dysfunction, oxidative stress, and dysregulated vasoactive mediators, is highlighted. Early detection and intervention during the prediabetic stage may reduce respiratory complications associated with T2DM, emphasizing the importance of management strategies targeting blood glucose regulation and vascular health. More research that looks into the mechanisms underlying pulmonary complications in T2DM and prediabetes is needed.

## 1 Introduction

Type 2 diabetes mellitus (T2DM) remains a substantial global health concern, with its prevalence escalating together with modern lifestyles characterized by sedentary habits and poor dietary choices (1). T2DM has long been known to have systemic effects that go beyond glycemic control, affecting several organ systems and subjecting patients to a wide variety of complications (2). T2DM is characterized by chronic hyperglycemia resulting from insulin resistance (1, 2). With its prevalence steadily rising worldwide, T2DM not only poses considerable burden on the healthcare system but also brings substantial morbidity and mortality risks to affected individuals (2). Unlike type 1 diabetes, which typically manifests in childhood or adolescence and involves autoimmune destruction of pancreatic beta cells, T2DM predominantly affects adults, and it is strongly associated with lifestyle factors such as obesity, physical inactivity, and an unhealthy diet (1, 3).

The pathogenesis of T2DM involves a complex interaction of genetic, environmental, and metabolic factors (4). Insulin resistance, a hallmark feature of T2DM, arises from impaired insulin signaling in peripheral tissues such as skeletal muscle, liver, and adipose tissue (5). In T2DM, the skeletal muscle, liver, and adipose tissue cells cannot respond to insulin as they should (5, 6). This can be due to insulin receptor resistance that develops over time, mainly from an inactive lifestyle and diet high in carbohydrates (5). This means blood glucose uptake by cells cannot occur due to impaired signaling resulting from the ineffective binding of insulin to its receptors (6). The impaired insulin signaling leads to a diminished uptake of blood glucose, resulting in chronic hyperglycemia and the clinical manifestation of diabetes (6). Beyond its well-established associations with cardiovascular disease, nephropathy, and neuropathy, growing evidence suggests that T2DM may also exert profound effects on pulmonary health and function (7, 8). Emerging research indicates a potential link between T2DM and pulmonary vascular dysfunction, characterized by alterations in pulmonary vascular tone, endothelial dysfunction, and increased risk of pulmonary hypertension (9, 10). Moreover, T2DM has been implicated in the pathogenesis of pulmonary fibrosis, a progressive lung disorder characterized by aberrant wound healing, fibroblast activation, and excessive extracellular matrix deposition in the pulmonary interstitium (10).

The onset of T2DM has been shown to be preceded by an intermediate hyperglycemic condition known as prediabetes (11, 12). Prediabetes is an asymptomatic state where the blood glucose concentration is greater than average but remains below the diagnostic threshold of T2DM (11). Studies have reported that T2DM-related disorders begin during prediabetes (11–13). The review aims to discuss the relationship between T2DM, pulmonary vascular dysfunction, and pulmonary fibrosis, with a specific focus on exploring potential links with prediabetes.

## 2 Overview of pulmonary vasculature and its function

Gaseous exchange in the lungs is made more accessible by the unique circulatory system of the pulmonary vascular system (14, 15). Its physiology and anatomy have been precisely tuned to maximize carbon dioxide and oxygen exchange while preserving the proper flow of blood and pressure (14). The pulmonary arteries receive deoxygenated blood from the heart’s right ventricle and pump it to the lungs, where it is oxygenated (15). As the pulmonary arteries get closer to the alveoli, they gradually divide into arterioles and capillaries (16). Effective gas exchange between alveolar air and blood is made possible by the thin walls of the pulmonary capillaries and alveoli (15). Oxygen diffuses from the alveoli into the capillaries, where it binds to hemoglobin in red blood cells, while carbon dioxide diffuses in the opposite direction, from the blood into the alveoli, to be exhaled (15, 17). Unlike the systemic circulation, the pulmonary circulation operates at much lower pressures (18). The mean pulmonary artery pressure (mPAP) is typically around 12–16 mmHg at rest, compared to around 70–120 mmHg in the systemic circulation (16). Dysfunction in the abovementioned processes can lead to pulmonary vascular diseases such as pulmonary hypertension, impair gas exchange and compromise respiratory function (18).

The pulmonary vascular system is under the control of various physiological mechanisms that regulate blood flow and pressure (16). Alveolar oxygen tension, carbon dioxide levels, and pulmonary vascular resistance (PVR) influence pulmonary blood flow (18, 19). PVR refers to the resistance to blood flow in the pulmonary circulation (19). It is primarily determined by the degree of constriction or dilation of the pulmonary arterioles and small arteries, which are regulated by factors like oxygen tension, endothelial-derived vasodilators such as nitric oxide (NO) and prostacyclin (PGI2), and vasoconstrictors such as endothelin-1 (ET-1) and thromboxane A2 (TxA2) (20–22). Thrombospondin-1 (THBS1), a matricellular protein, also plays a crucial role by regulating vascular cell interactions and influencing the extracellular matrix (23, 24). These mechanisms collectively maintain relatively low pulmonary arterial pressure compared to systemic arterial pressure, facilitating efficient oxygenation of blood in the lungs without imposing undue stress on the pulmonary vasculature (20, 21).

### 2.1 The role of nitric oxide and prostacyclin in the pulmonary vascular system

In endothelial cells, NO is produced by endothelial nitric oxide synthase (eNOS) and is released in response to various stimuli, including shear stress and vasoactive substances (25, 26). NO diffuses into nearby smooth muscle cells in the pulmonary vasculature, activating guanylate cyclase and producing cyclic guanosine monophosphate (cGMP) (27). The cascade of events results in the relaxation of vascular smooth muscle cells, vasodilation, and, ultimately, a decrease in pulmonary vascular resistance (27). NO also inhibits platelet activation and adhesion to endothelial surfaces, thus ensuring that blood vessels remain open and unobstructed (28, 29). Additionally, NO has anti-inflammatory properties and modulates endothelial cell function, contributing to the overall health and integrity of the pulmonary vasculature (27, 30). NO and PGI2 play crucial roles in maintaining normal pulmonary vascular function by promoting vasodilation and inhibiting platelet aggregation, among other functions (30, 31).

PGI2 is synthesized by endothelial cyclooxygenase (COX) in the endothelial cells and acts as a potent vasodilator and inhibitor of platelet aggregation (32). Like NO, PGI2 signals through cyclic adenosine monophosphate (cAMP) to induce relaxation of vascular smooth muscle cells and vasodilation (33). PGI2 also antagonizes the effects of vasoconstrictors such as ET-1 and TxA2, further promoting vasodilation and maintaining vascular tone (21, 33). Moreover, prostacyclin exerts anti-inflammatory and anti-thrombotic effects, contributing to the overall homeostasis of the pulmonary circulation.

NO and PGI2 act synergistically to regulate pulmonary vascular tone, promote vasodilation, inhibit platelet aggregation, and maintain vascular homeostasis (21). Dysregulation of NO and PGI2 pathways can lead to endothelial dysfunction, vasoconstriction, and increased vascular resistance, contributing to the pathogenesis of pulmonary vascular diseases such as pulmonary hypertension (21, 33). Therefore, preserving the production and signaling of nitric oxide and prostacyclin is essential for preserving normal pulmonary vascular function and preventing vascular pathology (34).

### 2.2 The role of endothelin-1 and thromboxane A2 in the pulmonary vascular system

ET-1 is a potent vasoconstrictor peptide produced primarily by endothelial cells (35). In pulmonary circulation, ET-1 contributes to regulating vascular tone by causing the vascular smooth muscle cells in pulmonary arterioles to contract (36, 37). Under normal conditions, ET-1 levels are tightly regulated to prevent excessive vasoconstriction, ensure appropriate pulmonary blood flow, and maintain sufficient arterial oxygenation (37). However, dysregulation of ET-1 can result in pulmonary hypertension and elevated pulmonary vascular resistance in several pathological conditions (37, 38). In addition to its vasoconstrictive properties, ET-1 also plays a role in triggering inflammatory responses and promoting the proliferation of endothelial cells, both of which can affect the remodeling of the pulmonary vascular system and disease progression (37). ET-1 and TxA2 are vasoactive substances that are essential in regulating pulmonary vascular function, although they primarily exert vasoconstrictive effects (38).

TxA2 is a lipid mediator primarily produced by platelets, and it is obtained from the metabolism of arachidonic acid with the help of COX and thromboxane synthase (TxAS) (39). TxA2 promotes thrombosis and vascular smooth muscle contraction in the pulmonary vasculature by acting as a strong vasoconstrictor and platelet aggregator (39). Similar to ET-1, TxA2 has a role in maintaining hemostasis and pulmonary vascular tone in normal physiological conditions (39). On the other hand, unregulated TxA2 signaling, which is frequently linked to endothelial dysfunction and inflammation, can cause excessive vasoconstriction, platelet activation, and blood clotting incidents in the pulmonary circulation (40, 41). In the pulmonary vascular system, ET-1 and TxA2 both essentially have vasoconstrictive actions, although their exact roles extend beyond simple vasoconstriction (40). Understanding the interplay of these vasoactive mediators is crucial for hopefully understanding the pathophysiology of pulmonary vascular diseases and developing targeted therapeutic interventions to restore normal pulmonary vascular function.

**Figure 1:**
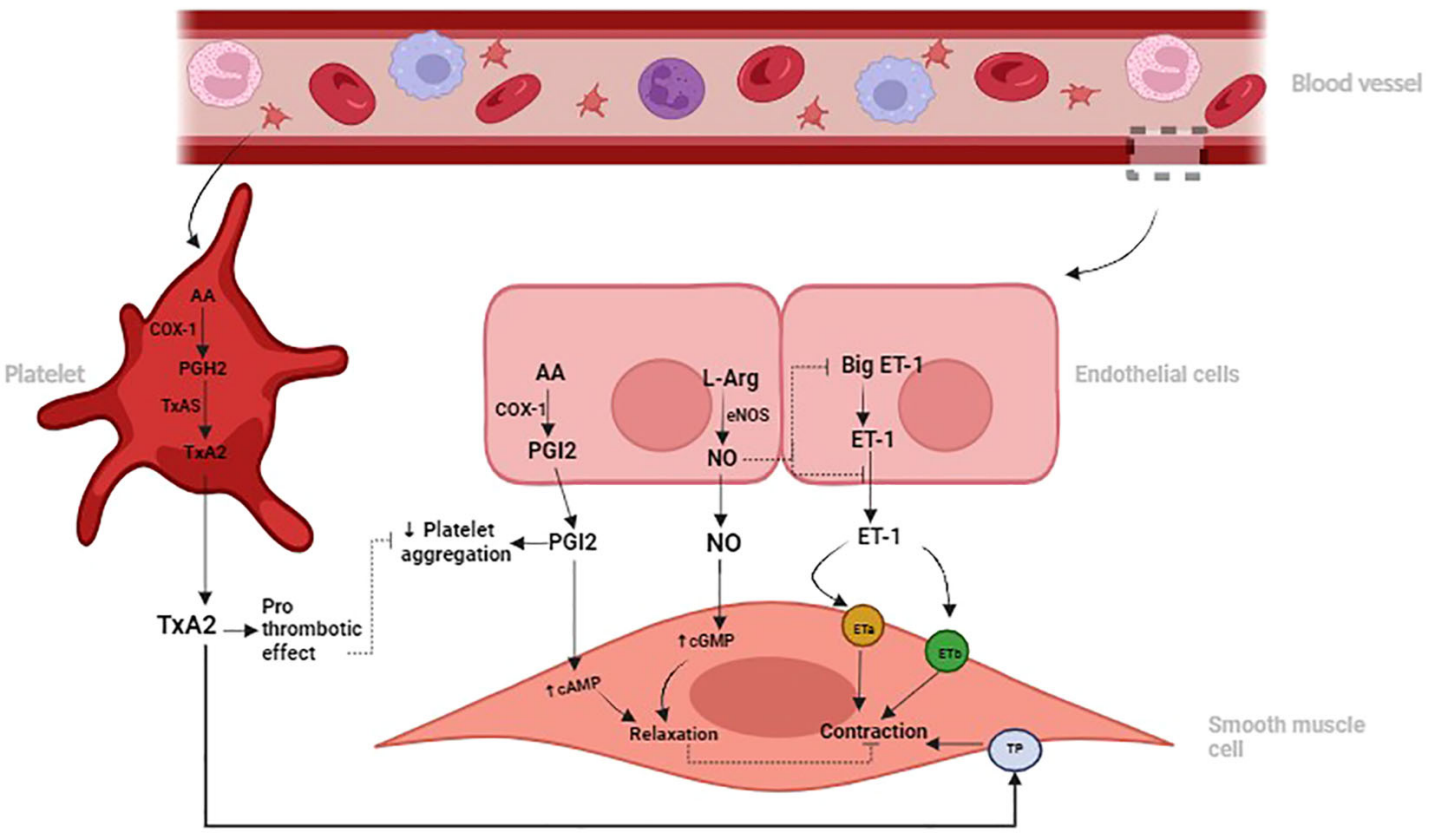
Illustration depicting the roles of Nitric Oxide (NO) and Prostacyclin (PGI2) in promoting vasodilation and inhibiting platelet aggregation, contrasting with the vasoconstrictive and prothrombotic effects of Endothelin-1 (ET-1) and Thromboxane A2 (TxA2) in the pulmonary vascular system. Dysregulation of these signaling pathways can contribute to pulmonary vascular diseases such as pulmonary hypertension and fibrosis.

Increased ET-1 and TxA2 concentrations promote vascular constriction, inflammation, and fibroblast activation, thereby exacerbating fibrotic processes in the lung (38, 41). Conversely, NO and PGI2 are vasodilators with anti-inflammatory and anti-fibrotic effects, counteracting the actions of ET-1 and TxA2 (42). NO and PGI2 inhibit fibroblast proliferation, collagen deposition, and myofibroblast differentiation, reducing pulmonary fibrotic remodeling (21, 33). Dysregulation of the balance between these vasoactive mediators, characterized by decreased NO and PGI2 and increased ET-1 and TxA2, contributes to the development and progression of pulmonary fibrosis (43). See **Figure 1** below.

### 2.3 The role of thrombospondin-1 in the pulmonary vascular system

The THBS1 is a protein that plays a significant role in regulating pulmonary vascular function and remodeling (23). THBS1 is produced by different cell types, including endothelial cells, smooth muscle cells, and fibroblasts, and is involved in cell-matrix interactions, angiogenesis, and tissue remodeling (23, 24). In the pulmonary vasculature, THBS1 regulates endothelial cell function, vascular smooth muscle cell proliferation, and extracellular matrix deposition (24, 44). THBS1 can inhibit NO signaling by binding to and activating CD36 and CD47 receptors on endothelial cells, leading to a decrease NO bioavailability and increased vascular resistance (24, 44). Additionally, THBS1 can promote the activation of latent transforming growth factor-beta (TGF-β), a key mediator of fibrosis and extracellular matrix deposition (44). Elevated THBS1 levels have been associated with pulmonary hypertension, fibrosis, and other vascular diseases, highlighting its role in pulmonary vascular dysfunction (24).

## 3 Overview of pulmonary fibrosis

Pulmonary fibrosis is a group of interstitial lung diseases characterized by progressive scarring (fibrosis) of the lung tissue (43). The scarring thickens and stiffens the lung walls, which hinders the circulatory system’s capacity to receive oxygen (43). There are several kinds of pulmonary fibrosis, the most common and prevalent among them being idiopathic pulmonary fibrosis (IPF) (45).

Additional subgroups include drug-induced interstitial lung disease, hypersensitivity pneumonitis, and connective tissue disease-associated interstitial lung disease (CTD-ILD) (46). Several variables, including exposure to pollutants, drug toxicity, autoimmune disorders, infections, and genetic predispositions, can cause pulmonary fibrosis (47). Older age, male gender, smoking, specific employment, and a family history of interstitial lung disease are risk factors for developing pulmonary fibrosis (48). While the exact cause of idiopathic pulmonary fibrosis remains unknown, a combination of genetic susceptibility and environmental triggers is thought to contribute to its development (48, 49).

Complex interactions between immune cells, fibroblasts, extracellular matrix, and epithelial cells are part of the pathophysiology of pulmonary fibrosis (47). Chronic damage to the alveolar epithelium results in abnormal wound healing responses marked by excessive collagen deposition, dysregulated inflammation, and fibroblast activation (49). These reactions are frequently brought on by recurring microinjuries or environmental exposures (49). This impairs gas exchange and lung function by causing fibrotic foci to develop and gradual scarring inside the lung parenchyma (49). The lungs’ fibrotic remodeling is sustained by defective repair mechanisms, poor apoptotic cell clearance, and abnormal activation of profibrotic signaling pathways including Wnt/β-catenin and TGF-β (50). Additionally, THBS1 plays a significant role in the pathogenesis of pulmonary fibrosis (23). THBS1, a matricellular protein, is involved in the regulation of cellular interactions and the extracellular matrix and it mediates TGF-β activation, contributing to fibrotic remodeling and progression of the disease (51).

Pulmonary fibrosis diagnosis is a complex process that includes imaging assessments, pulmonary function testing, clinical assessment, and invasive procedures for final diagnosis and monitoring of the disorder’s progression (49). High-resolution computed tomography (HRCT) imaging, which enables the identification of distinctive radiological characteristics such as reticular opacities, honeycombing, and traction bronchiectasis inside the lung parenchyma, is one of the critical criteria tested to diagnose pulmonary fibrosis (10, 49). The assessment of lung function and the severity of fibrotic lung disease is primarily dependent on pulmonary function tests (PFTs), which measure parameters including forced vital capacity (FVC), forced expiratory volume in one second (FEV1), and diffusing capacity of the lungs for carbon monoxide (DLCO) (49).

Restrictive lung physiology is frequently observed in pulmonary fibrosis, and it is indicated by reduced FVC and DLCO as well as a maintained or increased FEV1/FVC ratio (52). Furthermore, arterial blood gas measurement aids in determining the degree of reduced oxygen levels, respiratory failure, and gas exchange efficiency (53). In some cases, the bronchoalveolar lavage (BAL) or surgical lung biopsy may gather tissue samples for histological examination, which is still the gold standard for diagnosing pulmonary fibrosis and differentiating it from other interstitial lung diseases (54). Surfactant protein-D (SP-D) and Krebs von den Lungen-6 (KL-6) serum levels are examples of biomarkers that may offer further insights into the prognosis and progression of the disease (55). Proper diagnosis generally depends on a thorough assessment of clinical, radiological, functional, and histological criteria.

### 3.1 Exploring the interplay between T2DM and pulmonary vascular function

T2DM is a chronic metabolic disorder characterized by insulin resistance and impaired glucose metabolism that can cause systemic complications that impact several organs, including the lungs (2). Findings suggest that T2DM may significantly impact the pulmonary vasculature and systemic vasculature, which may aid in developing pulmonary vascular dysfunction (34, 56).

Research on the epidemiology of diabetes mellitus has demonstrated a strong association between T2DM and pulmonary vascular disorders, including pulmonary arterial stiffness and pulmonary hypertension (34). Pulmonary vascular dysfunction, characterized by endothelial dysfunction, vascular remodeling, and decreased pulmonary vascular reactivity, is more common in people with T2DM (56). One of the main characteristics of T2DM is endothelial dysfunction, defined by decreased NO bioavailability, impaired vasodilation, and increased endothelial permeability (57). Oxidative stress can reduce NO bioavailability by promoting the formation of peroxynitrite, a reactive nitrogen species that inactivates NO and damages endothelial cells (58). Additionally, oxidative stress can enhance the expression and activity of ET-1, TxA2, and THBS1, further contributing to vasoconstriction, endothelial dysfunction and vascular remodeling (58, 59). These factors cause abnormal pulmonary vascular tone and responsiveness (57).

Physiologically, endothelial dysfunction and vascular remodeling in the pulmonary circulation are caused by hyperglycemia-induced oxidative stress, inflammation, and dysregulated insulin signaling pathways (34, 57). Reactive oxygen species (ROS), proinflammatory cytokines, and advanced glycation end products (AGEs) increase smooth muscle growth, fibrotic remodeling, and vascular oxidative damage in the pulmonary vasculature, aggravating pulmonary vascular dysfunction in people with T2DM (60, 61).

Furthermore, by encouraging vasoconstriction, platelet activation, and vascular inflammation, metabolic dysregulation in T2DM, which includes dyslipidemia, hyperinsulinemia, and insulin resistance, increases pulmonary vascular dysfunction (62). The buildup of lipids and cholesterol in the pulmonary arteries caused by dyslipidemia impairs pulmonary vascular compliance and function by causing atherosclerosis, arteriosclerosis, and pulmonary vascular stiffness (62). Exercise intolerance, dyspnea, and decreased exercise capacity are clinical manifestations of pulmonary vascular dysfunction in people with T2DM, which resembles symptoms of pulmonary arterial hypertension (PAH) (63). Due to symptoms that might be confused with those of other cardiopulmonary disorders, diagnosing pulmonary vascular dysfunction in patients with T2DM is complicated (63). This highlights the importance of a thorough cardiovascular examination and interdisciplinary therapeutic strategies.

#### 3.1.1 Nitric oxide and prostacyclin in T2DM

In individuals with type T2DM, changes in the PGI2 and NO signaling pathways are linked to endothelial dysfunction and impaired pulmonary vascular function (64). Due to decreased eNOS activity, impaired endothelial NO synthase coupling, and hyperglycemia-induced elevated oxidative stress, there is a reduced bioavailability of NO in T2DM (61). This contributes to pulmonary vascular remodeling and hypertension in the pulmonary vasculature by causing endothelial dysfunction, vasoconstriction, and increased vascular smooth muscle growth (61).

In individuals with T2DM, dysregulation of prostacyclin synthesis and signaling pathways occurs due to various mechanisms, including impaired endothelial function, oxidative stress, and inflammation (61). Reduced bioavailability of prostacyclin, often accompanied by an imbalance with vasoconstrictor mediators like TXA2, disrupts the delicate equilibrium of vasodilation and vasoconstriction, leading to endothelial dysfunction and vascular remodeling (65). Additionally, hyperglycemia-induced activation of protein kinase C (PKC) and increased production of ROS inhibit prostacyclin synthesis and promote vascular oxidative damage, exacerbating vascular dysfunction in T2DM (66).

#### 3.1.2 Endothelin-1 and thromboxane A2 in T2DM

In individuals with T2DM, dysregulated endothelial function and increased vascular tone are mediated by various molecular pathways, among which ET-1 and TxA2 play pivotal roles (67). In T2DM, oxidative damage and inflammation triggered by hyperglycemia cause ET-1 to be excessively expressed (67). Increased PVR and compromised pulmonary hemodynamics result from endothelial dysfunction and vascular remodeling in the pulmonary circulation, which are facilitated by elevated ET-1 levels (21). Similarly, TxA2 contributes to pulmonary vascular dysfunction in T2DM by promoting platelet aggregation, inflammation, and vasoconstriction (58). Disrupting vascular homeostasis caused by dysregulation of the ET-1/ET receptor and TxA2/prostaglandin pathways leads to pulmonary artery construction and remodeling, significant indicators of pulmonary vascular disorders in people with T2DM (58, 68).

#### 3.1.3 Thrombospondin-1 in T2DM

Chronic high blood glucose concentration in T2DM result in the production of AGEs, stimulating THBS1 expression via the receptor for AGEs (RAGE) and downstream pathways (69, 70). Insulin resistance in T2DM disrupts metabolic signaling, impairing the regulatory effects of insulin on THBS1 and promoting inflammation, further increasing THBS1 concentration (62). The chronic low-grade inflammation in T2DM involves elevated TNF-α and IL-6, inducing THBS1 expression through activation of nuclear factor kappa B (NF-κB) (69, 71). Oxidative stress activates pathways like mitogen-activated protein kinases (MAPKs), driving THBS1 transcription (71). Endothelial cells exposed to hyperglycemia and oxidative stress produce more THBS1, inducing vascular damage (72). This multifactorial increase in THBS1 plays a crucial role in T2DM vascular complications, including endothelial dysfunction, vascular remodeling, and increased risk of thrombosis (73).

THBS1 interacts with receptors like CD36, integrins, and CD47, activating pathways that promote endothelial cell apoptosis, inhibit angiogenesis, and reduce NO bioavailability (74, 75). By binding to its receptors, THBS1 inhibits the activation of eNOS, reducing NO production (74). NO deficiency leads increases vascular tone and endothelial dysfunction, hallmark of the vascular complications in T2DM (76). Reduced NO impairs vasodilation, increasing vascular resistance and hypertension (73). Elevated THBS1 in T2DM activates TGF-β, contributing to vascular remodeling, fibrosis, and stiffness, promoting a pro-fibrotic environment, and advancing complications (77, 78).

THBS1 also facilitates macrophage recruitment and activation, releasing pro-inflammatory cytokines and ROS, damaging the endothelium, and promoting atherosclerosis (70, 73). Oxidative stress from THBS1-mediated inflammation leads to endothelial cell injury and dysfunction, perpetuating vascular damage (79). THBS1, released from activated platelets, promotes aggregation and adhesion to the endothelium, increasing vascular occlusions and cardiovascular event risk in T2DM (79, 80). By enhancing platelet activation and promoting a pro-coagulant state, THBS1 increases the vascular complications associated with diabetes (80).

Together, these processes induce vascular stiffness, intensify atherosclerosis, and increase the risk of thrombotic events, all of which are important in the development of severe cardiovascular complications associated with T2DM (62, 78, 79).

### 3.2 Mechanisms linking T2DM to pulmonary fibrosis

Findings have shown that T2DM can significantly impact several organ systems, including the lungs (81). The complicated mechanisms by which T2DM aids in the onset and progression of pulmonary fibrosis, a crippling lung condition marked by an overabundance of extracellular matrix protein deposition and tissue remodeling, have been clarified by recent studies (81, 82). One well-known mechanism is the interaction of oxidative stress, dysregulated fibrotic signaling cascades, and chronic inflammation in the pulmonary environment (81, 82). The production of ROS and proinflammatory cytokines is enhanced by T2DM-associated hyperglycemia and dyslipidemia, which in turn causes inflammatory reactions and cellular damage in lung tissues (60). Profibrotic pathways, such as the TGF-β signaling pathway, encourage fibroblast activation and collagen deposition is subsequently activated by these inflammatory mediators (60, 83). Furthermore, abnormal vascular remodeling and dysregulated lipid metabolism lead to the buildup of AGEs and endothelial dysfunction in the pulmonary vasculature, exacerbating lung fibrotic modifications (84). The multifaceted character of this disease process is further demonstrated by the possibility that interactions between fibrogenic signaling cascades and metabolic pathways could speed up the development of pulmonary fibrosis in T2DM patients (21, 84).

**Figure 2:**
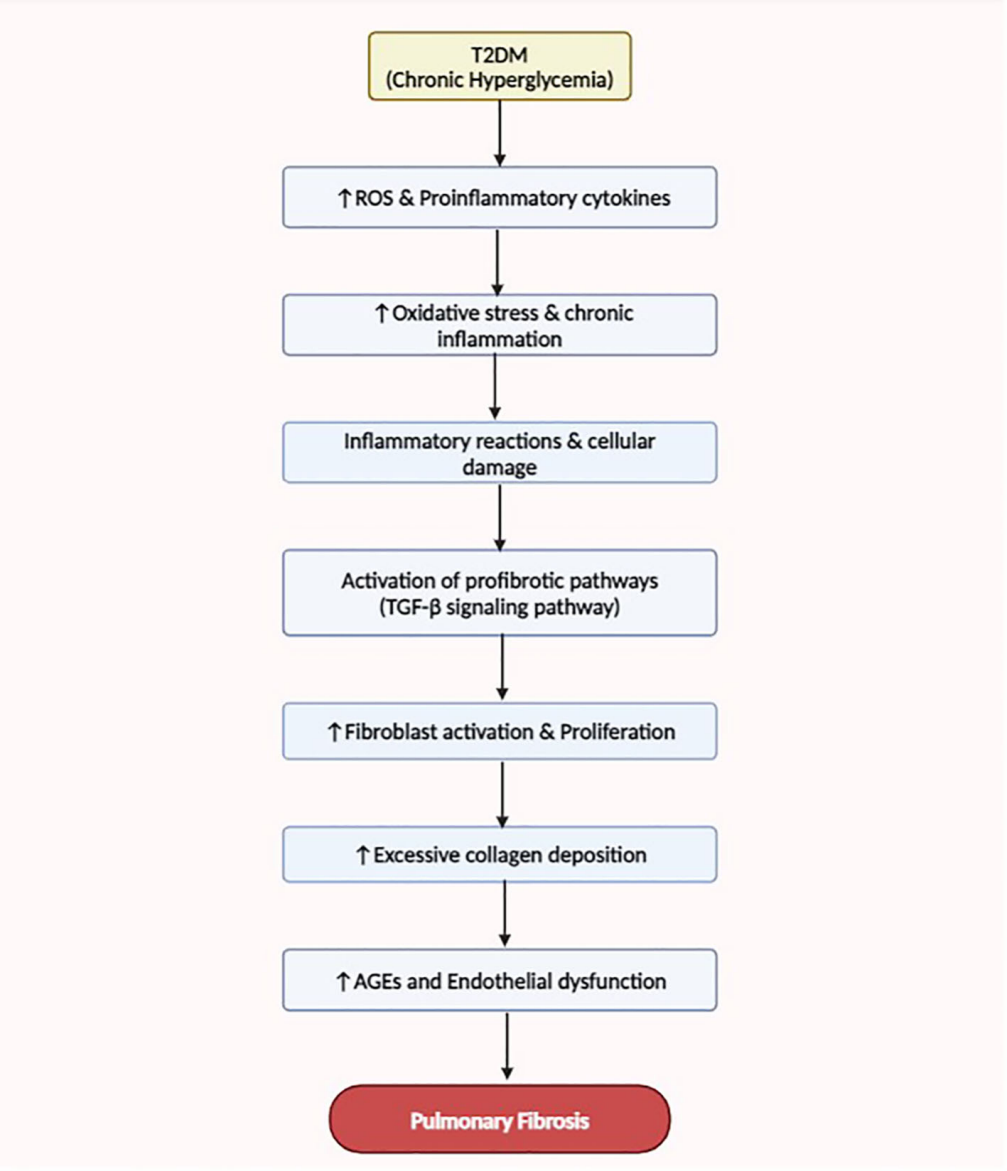
This flow diagram illustrates the mechanisms linking T2DM to the development and progression of pulmonary fibrosis. Enhanced oxidative stress, dysregulated fibrotic signaling, chronic inflammation, and abnormal vascular remodeling contribute to the pathogenesis of lung fibrosis in T2DM patients.

Recent research indicates that complications related to T2DM often manifest during the prediabetic state, underscoring the significance of early detection and intervention (11, 12). There is growing evidence that respiratory disorders may also be associated with prediabetes (11–13). While the primary focus has traditionally been on the risk of cardiovascular disease and microvascular complications in prediabetes, attention should also be increased to the potential impact on pulmonary health (85). See in **Figure 2** below.

## 4 Prediabetes

Prediabetes is a metabolic condition that serves as an intermediate stage in the progression from normal glucose metabolism to overt diabetes and is associated with an increased risk of developing T2DM and cardiovascular disease (85, 86). This intermediate hyperglycemic state is characterized by impaired glucose tolerance and elevated blood sugar levels below the threshold for diabetes diagnosis (86). Prediabetes encompasses two fundamental conditions: impaired glucose tolerance (IGT), which occurs when blood glucose levels increase above normal after an oral glucose tolerance test (OGTT), and impaired fasting glucose (IFG), which occurs when fasting blood glucose levels increase above average but not diagnostic of T2DM (87). Prediabetes is diagnosed based on specific threshold values for 2-hour plasma glucose (2-h PG) and fasting plasma glucose (FPG) levels obtained following an OGTT (88). Obesity, a sedentary lifestyle, a family history of diabetes, and specific ethnic origins are risk factors for prediabetes. Although prediabetes has historically been linked to abnormalities in systemic metabolism and a higher risk of cardiovascular disease, new research indicates that lung health may also be affected (87, 88). Risk factors for prediabetes include obesity, sedentary lifestyle, family history of diabetes, and specific ethnic backgrounds (88). While prediabetes has traditionally been associated with systemic metabolic disturbances and an increased risk of cardiovascular disease, emerging evidence suggests potential implications for pulmonary health as well (7, 13).

### 4.1 Possible links of prediabetes to the pulmonary system

Prediabetes shares standard pathophysiological features with T2DM, including insulin resistance, dyslipidemia, and chronic low-grade inflammation, which can exert detrimental effects on the pulmonary vasculature and lung parenchyma (89, 90). Emerging evidence suggests a potential interconnection between prediabetes and pulmonary health, raising intriguing questions about the systemic impact of metabolic disturbances on respiratory function (13, 91–93). These studies have highlighted the direct effects of hyperglycemia and metabolic dysfunction on pulmonary endothelial cells and alveolar epithelial cells, contributing to endothelial dysfunction, pulmonary inflammation, oxidative stress, and lung fibrotic remodeling (91–93).

NO plays a crucial role in vascular health by regulating blood flow, tone, and endothelial function (90, 94). Research indicates that prediabetic conditions, such as insulin resistance and impaired glucose metabolism, disturb the delicate balance of NO production and bioavailability (90, 92). Insulin resistance can impair endothelial eNOS activity, reducing NO production (92, 93). The elevated blood glucose concentration associated with prediabetes can also promote oxidative stress and inflammation, which further deplete NO levels and impair NO-mediated signaling pathways (95). Consequently, decreased NO availability in prediabetes contributes to endothelial dysfunction, vasoconstriction, and impaired vascular relaxation, predisposing individuals to cardiovascular complications like hypertension, atherosclerosis, and endothelial dysfunction (92, 96).

In prediabetes, the THBS1 pathway could significantly aggravate these complications (97). Insulin resistance might disrupt insulin signaling, which would increase the concentration of THBS1 and worsen endothelial dysfunction and inflammation (23, 75, 97). Elevated blood glucose in prediabetes could lead to the formation of AGEs, which might bind to the RAGE and upregulate THBS1 expression, further impairing endothelial function (51). THBS1 might inhibit eNOS, reducing NO production, resulting in vasoconstriction, and increased vascular resistance (69). Chronic low-grade inflammation, driven by cytokines like TNF-α and IL-6, could induce THBS1 expression through NF-κB activation, enhancing macrophage recruitment and perpetuating vascular inflammation (69, 72). Oxidative stress might activate signaling pathways like MAPKs that increase THBS1 levels, contributing to endothelial cell injury (9). Additionally, THBS1 could promote a pro-thrombotic environment by enhancing platelet aggregation and adhesion, increasing the risk of vascular occlusions and cardiovascular events (98).

The elevated blood glucose concentration in prediabetes can extensively affect PGI2, a vital molecule involved in vascular function (99, 100). As prediabetes progresses, hyperglycemia promotes a series of events leading to increased oxidative stress and inflammation, resulting in endothelial dysfunction, which impairs the production and release of PGI2 (101). Furthermore, AGEs can be formed due to hyperglycemia, further impairing PGI2 production and function. As a result, the reduction in PGI2 concentration contributes to endothelial dysfunction, vasoconstriction, platelet aggregation, and ultimately, an increased risk of cardiovascular complications associated with prediabetes (93, 101). Furthermore, this PGI2 dysregulation may serve as a crucial link between prediabetes and the development of overt T2DM and its complications, emphasizing the importance of early intervention and management strategies targeting both glycemic control and vascular health.

In a prediabetic state, increased insulin resistance and hyperglycemia lead to endothelial dysfunction, which manifests through elevated ET-1 levels (102). The increased ET-1 concentration is primarily mediated by the upregulation of endothelin-converting enzyme activity and increased production from endothelial cells (103, 104). Additionally, prediabetes promotes an imbalance in the prostaglandin-thromboxane axis, which promotes the production of TxA2 over PGI2, primarily due to modified platelet activation and arachidonic acid metabolism (105, 106). Increased TxA2 enhances inflammation, platelet aggregation, and vasoconstriction, which makes surrounding tissue more prone to atherosclerosis and thrombosis (107). Therefore, if left unchecked, the dysregulation of ET-1 and TxA2 in prediabetes promotes endothelial dysfunction, thrombosis, and vascular complications together, developing the conditions for the onset of overt T2DM and cardiovascular illnesses (102, 107).

The transition from prediabetes to overt T2DM may further intensify these pulmonary complications, highlighting the importance of early intervention and preventive measures (89). However, the precise mechanisms linking prediabetes to pulmonary pathology remain incompletely understood. Hence, more research is needed to look at the relationship between prediabetes and pulmonary dysfunction, including reduced pulmonary function, an elevated risk of respiratory infections, and maybe the onset of pulmonary fibrosis.

## 5 Conclusion

The complex relationship among prediabetes, T2DM and pulmonary disorders highlights the critical need for extensive research into prediabetes and its underlying mechanisms to mitigate the progression to overt T2DM. Complications such as pulmonary vascular dysfunction and fibrotic lung disease significantly increase morbidity, mortality and diminish the quality of life in individuals with T2DM. Addressing pulmonary disorders in prediabetic individuals can potentially prevent the progression to overt T2DM. Early identification and management of prediabetes, with a focus on preventing pulmonary complications, may help reduce the risk of developing T2DM. Additionally, understanding and treating pulmonary disorders in prediabetic patients can improve patient outcomes and reduce the burden of metabolic disorders. Therefore, studies targeting both prediabetes and pulmonary health are essential for mitigating the progression to T2DM and improving overall patient health.

## 6 Future studies

Additional research into the pulmonary problems linked to T2DM and prediabetes will take many different forms to improve clinical outcomes, identify novel targets for treatment, and understand the underlying processes. Methods that include data from clinical trials, patient samples, and preclinical models, especially those including prediabetes, must be used. Understanding the early pathophysiological modifications and pathways that result in pulmonary vascular dysfunction in people with T2DM requires using such an approach. These initiatives are essential to reducing the burden that prediabetes-related pulmonary problems place on impacted people and healthcare systems across the world.

## Author contributions

NM: Conceptualization, Investigation, Visualization, Writing – original draft, Writing – review & editing. ND: Investigation, Writing – review & editing. AS: Supervision, Validation, Writing – review & editing. AK: Conceptualization, Supervision, Validation, Writing – review & editing.
